# Evaluation of Biosynthesis, Accumulation and Antioxidant Activityof Vitamin E in Sweet Corn (*Zea mays* L.) during Kernel Development

**DOI:** 10.3390/ijms18122780

**Published:** 2017-12-20

**Authors:** Lihua Xie, Yongtao Yu, Jihua Mao, Haiying Liu, Jian Guang Hu, Tong Li, Xinbo Guo, Rui Hai Liu

**Affiliations:** 1 School of Food Science and Engineering, South China University of Technology, Guangzhou510641, China; 201520120463@mail.scut.edu.cn (L.X.); 201520120456@mail.scut.edu.cn (H.L.); 2 Crop Research Institute, Guangdong Academy of Agricultural Sciences, Guangzhou 510640, China; yuyongtao@gdaas.cn (Y.Y.); maoer08@163.com (J.M.); jghu2003@263.net (J.G.H.); 3 Key Laboratory of Crops Genetics Improvement of Guangdong Province, Guangzhou 510640, China; 4 Department of Food Science, Stocking Hall, Cornell University, Ithaca, New York, NY 14853, USA; tl24@cornell.edu

**Keywords:** sweet corn, vitamin E, gene expression, antioxidant activity, kernel development

## Abstract

Sweet corn kernels were used in this research to study the dynamics of vitamin E, by evaluatingthe expression levels of genes involved in vitamin E synthesis, the accumulation of vitamin E, and the antioxidant activity during the different stage of kernel development. Results showed that expression levels of *_Zm_HPT* and *_Zm_TC* genes increased, whereas *_Zm_TMT* gene dramatically decreased during kernel development. The contents of all the types of vitamin E in sweet corn had a significant upward increase during kernel development, and reached the highest level at 30 days after pollination (DAP). Amongst the eight isomers of vitamin E, the content of γ-tocotrienol was the highest, and increased by 14.9 folds, followed by α-tocopherolwith an increase of 22 folds, and thecontents of isomers γ-tocopherol, α-tocotrienol, δ-tocopherol,δ-tocotrienol, and β-tocopherol were also followed during kernel development. The antioxidant activity of sweet corn during kernel development was increased, and was up to 101.8 ± 22.3 μmol of α-tocopherol equivlent/100 g in fresh weight (FW) at 30 DAP. There was a positive correlation between vitamin E contents and antioxidant activity in sweet corn during the kernel development, and a negative correlation between the expressions of *_Zm_TMT* gene and vitamin E contents. These results revealed the relations amongst the content of vitamin E isomers and the gene expression, vitamin E accumulation, and antioxidant activity. The study can provide a harvesting strategy for vitamin E bio-fortification in sweet corn.

## 1. Introduction

Vitamin E is recognized as the most important lipid-soluble, chain-breaking antioxidant in the human body, whose deficiency may lead to neurodegenerative diseases, such as Alzheimer’s disease, cystic fibrosis, and autosomal recessive ataxias [1]. Researchers found that the combined use of vitamin E and vitamin C supplements is associated with the reduced prevalence and incidence of Alzheimer’s disease [2]. The group of vitamin E family consists of tocopherols(T) and tocotrienols(T3), both of which have four isomers: α-, β-, γ-, and δ-forms, according to the number and position of methyl groups at the chromanol ring system, and they are collectively known as tocochromanols or tocols [3]. It is generally agreed that all tocochromanols are potent antioxidants with lipoperoxyl radical scavenging activities. Regarding vitamin E, many researches have mainly focused on the tocopherols, especially the α-T and γ-T, because α-T is the major vitamin E isomer in vivo, and it exerts the highest biological activity. Also, α-T is the predominant form of vitamin E in tissues, and an insufficient intake of this form can result in a vitamin E deficiency-associated ataxia [4]. Some studies, however, have found that γ-T was more potent than α-T in regard to certain biological functions, like the prevention of occlusive thrombus formation [5]. In plants, tocopherols play a role in the protection of the photosynthetic apparatus, and may affect gene expression. Different tocopherol homologues have special functions [3]. Moreover, there has been an increase in research focusing on the functions of tocotrienols in recent years. Those studies reveals that tocotrienols possess powerful neuroprotective, antioxidant, anti-cancer, and cholesterol lowering properties, which often differed from the properties of tocopherols [6]. At nanomolar concentration, α-T3, instead of α-T, prevents neurodegeneration [7]. Some findings indicated that tocotrienols were promising anticancer agents for minimizing tumor angiogenesis [8]. Both δ-T3 and (to a lesser extent) γ-T3 were more active than α-T and α-T3 in diminishing diet-induced metabolic syndrome in rats, hence increasing the intake of γ-T3, and δ-T3 may serve as a complementary dietary strategy in managing this syndrome [9].

Tocochromanols are composed of two distinct classes (including tocopherols and tocotrienols). They are synthesized from precursors which were derived from both the shikimate and the 2-C-methyl-d-erythritol-4-phosate MEP pathways of the inner chloroplast membrane in higher plants [10,11,12]. There are five key enzymes involved in biosynthesis of tocochromanol: *p*-hydrophenylpyruvate dioxygenase (HPPD), homogentisate phytyltransferase (HPT), 2-methyl-6-phytyl-1,4-benzoquinone methyltransferase (MPBQ-MT), tocopherol cyclase (TC), homogentisate geranylgeranyl transferase (HGGT), and γ-tocopherol methyltransferase (γ-TMT). Shikimate delivered *p*-hydroxyphenylpyruvate (HPP) to homogentisic acid (HGA) by HPPD. HGA was then prenylated with either phytyl diphosphate (PDP) or geranylgeranyl diphosphate (GGDP) (both from the MEP pathway) to produce 2-methyl-6-phytylbenzoquinol (MPBQ) or 2-methyl-6-geranylgeranyl benzoquinol (MGGBQ), in order to yield tocopherols and tocotrienols, respectively. In general, three subsequent activities, two methyltransferases and tocopherol cyclase (encoded by the *MPBQ/MGGBQ-MT*, *TMT*, and *TC*, respectively) regulated and generated the full chemical diversity of tocochromanols (α-, β-, δ-, and γ-tocopherols and tocotrienols). The cyclization completed by the TC could produce δ-T/T3, γ-T/3, respectively, and γ-TMT could methylate δ-T/T3 and γ-T/3 into β-T/T3 and α-T/T3. In maize, *TMT* and *HPPD* were found to show the co-localization with major quantitative trait loci (QTL) for tocopherol content and composition in maize grains [13].

Maize kernels have been studied for their different composition of vitamin E. Vitamin E is a relatively abundant compound in corn, especially the γ-tocopherol isomer, which occupies a large ratio of the total vitamin E. Phinil detected seven vitamin E isomers, except the β-tocotrienol, in sweet corn [14], and Ibrahim found that γ-tocopherol was the primary compound in sweet corn [15]. Since vitamin E is important to human health, animal growth, and plant maturity, the vitamin E contentin grains has attracted researchers’ attention [16]. Rather than using expensive genetic engineering approaches to increase the tocopherol concentrations, breeding maize with high levels of tocopherols in the grain may provide an alternative source of vitamin E. Sweet corn is a naturally appearing recessive genetic mutation product of field maize. Amongst the maize, kernels can store more sugar than field corn, and possess more nutritional and functional properties [17]. Nowadays, immature sweet corn has become increasing wide spread as a snack or vegetable in Asian countries [18].

So far, several studies have shown the relationship between several vitamin E forms and a number of physiological processes, including germination [19], kernel development [20], and genetic dissection of tocopherols by QTL [13]. However, there are few reports on the relationship between accumulation of vitamin E and their biosynthesis during silking time to mature sweet corn. Our hypothesis for this study was that during the sweet corn kernel development, vitamin E might accumulate due to enhancement of the transcript levels of genes in biosynthesis pathways, and its antioxidant activity might also increase. Therefore, the objective of this study was to detect the transcription levels of key encoding genes in vitamin E biosynthesis pathways, the accumulation of vitamin E, and its antioxidant activity during sweet corn kernel development.

## 2. Results and Discussion

### 2.1. Expression Analysis of Key Synthetic Genes

**Figure 1:**
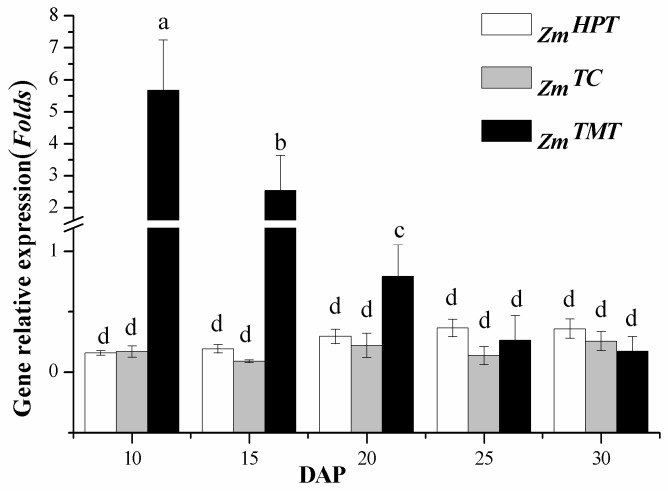
Relative expression of genes involved in vitamin E synthesis during sweet corn kernel development (mean ± SE). *_Zm_HPT* = homogentisate phytyltransferase; *_Zm_TC* = tocopherol cyclase; *_Zm_TMT* = γ-tocopherol methyltransferase; DAP = days after pollination. Bars with no letters in common are significantly different (*p* < 0.05).

Real-time PCR was conducted to analyze the variations of expression of genes encoding enzymes that participate in vitamin E synthesis inmaize, during sweet corn kernel development. As shown in Figure 1, the relative expression levels of *_Zm_HPT* increased and reached a plateau at 25 days after pollination (DAP), 2.25 times higher than the initial level at 10 DAP. For *_Zm_TC*, its expression levels remained relatively stable, with a small peak at 15 DAP. However, the highest *_Zm_TMT* expression were observed at 10 DAP, followed by a decrease at 15 DAP. The expression leveled off at the final stage of maturation. The result indicated that *_Zm_TMT* was much more active than *_Zm_HPT* and *_Zm_TC* during the sweet corn kernel development, and its expression levels were approximately 16 times higher than its expression levels at 10 DAP.

During the maturation of sweet corn, the storage products of the kernel are synthesized, beginning at around 15 DAP. The synthesis continues until the metabolic activity is prevented by desiccation at seed maturity (after 40 DAP) [21]. The expression levels of these three genes increased or decreased at the initial stage of sweet corn maturation (10–25 DAP), due to its vigorous growth period. This is followed by a gentle change during the late stage (25–30 DAP), because of a decline of metabolism, which was in coincidence with the maize kernel development rule. In this study, the expression levels of *_Zm_HPT* and *_Zm_TC* increased by 2.25 folds and 1.5 folds, respectively, whereas the level of *_Zm_TMT* decreased 33 folds at 10–30 DAP during the sweet corn kernel development. High expression of *_Zm_TMT* may result in the accumulation of the conversion of γ- to α-forms in the early developmental stage.

### 2.2. NP-HPLC Chromatogram and Peak Identification of Vitamin E

**Figure 2:**
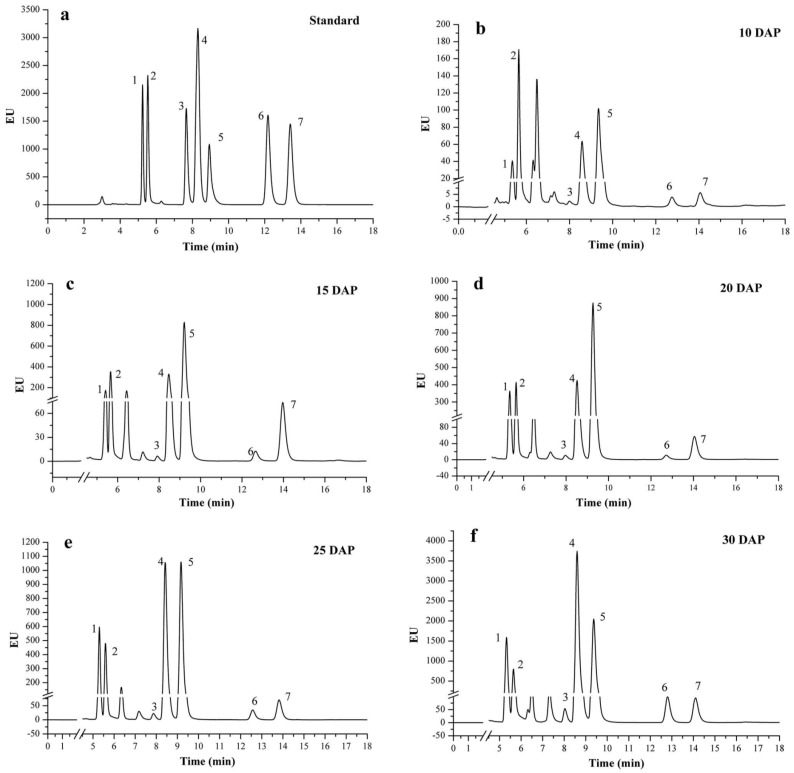
NP-HPLC chromatogram of vitamin E standard and extracts in sweet corn during kernel development (numbers 1–7 represent α-T, α-T3, β-T, γ-T, γ-T3, δ-T, δ-T3, respectively, T means tocopherol, and T3 means tocotrienol). (**a**) is the chromatograms of vitamin E standards; (**b**–**f**) are the chromatograms of sweet corn extracts from 10 DAP to 30 DAP. EU = Emission Unit.

An NP-HPLC system was used to separate all eight isomers and the internal standard. Figure 2 shows the chromatograms of vitamin E standards and sweet corn extracts during kernel development. A typical run would last for 20 min. By comparing the peaks with standard compounds, peak 1 to peak 6 of the chromatogram were identified as α-T, α-T3, β-T, γ-T, γ-T3, δ-T, and δ-T3, respectively. From the chromatogram, it showed that the response value of the vitamin E isomers increased as the days after pollination grows, especially the α-T and γ-T. However, the β-T, δ-T, and δ-T3 changed slightly. In our study, we were not able to quantify the β-T3 in the maize as well as in the mixture of standard compounds, because γ-T and β-T3 have a similar retention time on isocratic elution with the mobile phase (*n*-hexane/isopropanol). Moreover, the contents of β-T3 are low in sweet corn, and it was hard to separate it from γ-T. Comparing with other studies, our results show good consistency with others [14,22].

### 2.3. Changes of Vitamin E Composition and Contents

**Figure 3:**
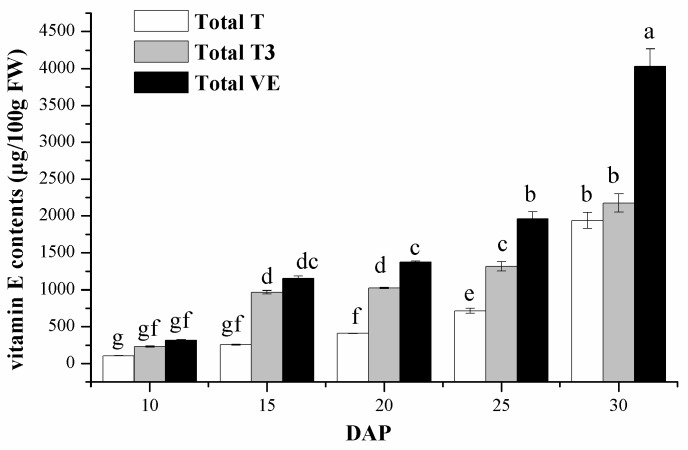
Total tocopherols (T), tocotrienols (T3) and vitamin E contents of sweet corn during kernel development (mean ± SD). Bars with no letters in common are significantly different (*p* < 0.05).

There is an obvious increase in the content of total T, total T3, and total vitamin E (*p* < 0.01) as shown in Figure 3. The contents of total T, T3, and total vitamin E contents are 106.7–1973 μg/100 g FW, 230.4–2176 μg/100 g FW, and 316.1–4033 μg/100 g FW, respectively, during the sweet corn kernel development.

According to Table 1, we found that α-T was the predominant component amongst total tocopherols with a ratio of 61%. And γ-T accounted for 28% amongst total tocopherols. Both the constitution of α-T and γ-T were the highest at 20 DAP. Interestingly, there was a study which showed that within 41 corn genotypes, the average total tocopherols contents were 54.1 μg/g dry weight at 21 DAP. γ-T accounted for 73% of total T, and α- and δ-T accounted for 24% and 3% of total T, respectively [15]. Most research mainly focuses on the tocopherols of sweet corn [20,23]; tocotrienols have received little attention, because they were widely known to be more predominant in corn oil (80 μg/g fresh weight) [15]. However, in our study, tocotrienols were shown to have a large proportion in sweet corn kernels, and accounted for 67.3% amongst total VE. The contents of tocotrienols ranked as γ-T3, α-T3, and δ-T3 (from highest to lowest).

The contents of all vitamin E isomers were reached to peaks at 30 DAP, especially α-T, γ-T, and γ-T3, which were 22-, 28.7-, and 14.8 folds higher than the initial amounts, respectively. For the α- and γ-isomers, the ratio of α-/γ- (sum of tocopherols and tocotrienols) was 0.96 at 10 DAP, and decreased at 15 DAP. Then, it reached a plateau at 25–30 DAP, the result was then the same as α-T3/γ-T3. The ratio of α-T/γ-T also declined at 10–15 DAP, but then increased at 15–20 DAP and went down again at 20–30 DAP. The ratio of T/T3 decreased at first, and then increased to the highest level at 30 DAP. The change of vitamin E composition and contents in sweet corn may result from the changing degree in expression of genes involved in vitamin E synthesis during the kernel natural maturation.

The overexpression of *HPT* and *TC* may cause tocopherol accumulation during plant growth. Harish and others found that the α-tocopherol content in transgenic tobacco plants was increased by 5.4-, 4.0-, and 7.1 folds with expressions of *HPT*, *TC*, and *HPT:TC*, respectively, when compared to the wild type [24]. Our results showed an agreement with this rule. The total tocopherol contents were increased by 18 folds in the sweet corn, due to the increasing expressions of *_Zm_HPT* and *_Zm_TC* during the kernel development, principally contributed to by α-T and γ-T. *TMT* is known to alter the vitamin E composition by converting δ- and γ-tocopherol (or tocotrienol) to β- and α-tocopherol (or tocotrienol), respectively. Overexpression of *TMT* leads to a complete conversion of *γ*-T to α-T in *Arabidopsis thaliana*, *Glycine max*, and *Zea mays* [25]. In our study, the *_Zm_TMT* had the highest expression level at 10 DAP, and then decreased and finally leveled off. Meanwhile, the ratio of β/δ-T (or T3) and α/γ-T (or T3) reached the highest level at 10 DAP, and was then maintained, while the ratio of α/γ-T fluctuated during the sweet corn kernel development (Table 1). The ratio of α- to γ- in tocopherol was higher than in tocotrienols. The result may reveal that TMT is more active in the conversion of γ-T to α-T, than γ-T3 to α-T3. The mechanism of TMT function in the tocopherol and tocotrienol synthesis needs further investigation.

During the sweet corn kernel development, tocotrienols were the predominant compounds in sweet corn. The major type of tocotrienol found in sweet corn is γ-T3 (with an averaged composition of 70%), while the major type of tocopherol was α-T (averaged composition of 50%). This result indicates that the conversion of γ- to α-form was active in tocopherol pathway, while the conversion was not completely active in tocotrienol synthesis. The result is similar to the grape seed development [26].

### 2.4. Changes in the Lipophilic Antioxidant Activity of the Sweet Corn Extracts

Vitamin E is a lipophilic antioxidant. All eight isomers have antioxidant activity, especially α-T, which is known as a strong chain-breaking and peroxyl radical-scavenging antioxidant, due to its 6-hydroxychroman structure [27]. Lipo-PSC assay was applied to measure the lipophilic antioxidant activity of sweet corn extracts (Table 2). This assay is based on the degree of inhibition of dichlorofluorescein oxidation caused by antioxidants that scavenge peroxyl radicals, which generated from thermal degradation of 2,2′-azobis [28]. The PSC values expressed as micromole of α-tocopherol equivalent per 100 g in fresh weight (μmol α-tocopherol equiv./100 g FW). In our study, the levels of lipophilic antioxidant activities between 10–15 DAP sweet corn were lower than the detection limits of PSC values. The PSC value of sweet corn was up to 39.08 ± 7.72 μmol α-tocopherol equiv./100 g FW at 20 DAP, and then the PSC value was increased significantly in a DAP-dependent manner, and reached the highest value (101.8 ± 22.3 μmol α-tocopherol equiv./100 g FW) at 30 DAP, approximately 2.6 folds compared with the value at 20 DAP.

## 3. Materials and Methods

### 3.1. Plant Material Preparation

YueTian-16 (YT-16), one of the most common varieties of sweet corn in South China that was used in this study, was provided by Crops Research Institute, Guangdong Academy of Agricultural Science. Fifteen YT-16 plants were selected and grown at the experimental farmland, with three biological replications in autumn. All plants were self-pollinated, and the dates of pollination were recorded. Three kernels per lines at 10, 15, 20, 25, and 30 DAP were harvested and immediately threshed. The threshed corn kernels were transported to our laboratory and stored at −20 °C before analysis. To obtain reliable samples, harvested samples were selected with uniform shape and size, without any physical damage or disease.

### 3.2. RNA Extraction and Quantitative Gene Expression Analysis

Total RNA from the developing kernels (10, 15, 20, 25, 30 DAP) was isolated using Plant RNA Kit (TIANGEN, Beijing, China) according to the manufacturer’s instructions. Then, the high-quality RNA samples were used to synthesize the first-strand cDNA sequences with PrimeScript^TM^ RT reagent Kit with gDNA Eraser (Takara Biotechnology, Dalian, China). The transcription levels of the genes involved in vitamin E synthesis during sweet corn kernel development were quantified by quantitative real time-PCR analysis using SYB^®^ Prime-Script RT-PCR kits (TIANGEN, Beijing, China) and finished by LightCycler^®^480 Real-Time PCR System (F. Hoffmann-La Roche Ltd., Basel, Switzerland). The cDNA sequences of putative genes involved in the tocopherol biosynthetic pathway in maize grain were obtained from NCBI (http://www.ncbi.nlm.nih.gov/). The key-encoding genes of vitamin E biosynthesis pathways in sweet corn were determined as followed: *_Zm_HPT* (Gene ID: 732789), *_Zm_TC* (Gene ID: 541877), and *_Zm_TMT* (Gene ID: 732837). The relative transcription levels of each gene in different RNA samples were normalized with respect to the internal standard *β-actin* gene (Gene ID: 100282267). *_Zm_Actin* is proved as a suitable reference gene for PCR in maize as reported previously [29,30,31]. Real-time primers were designed by Primer5 software (Primer Biosoft, Palo Alto, CA, USA) as Table 3. The thermal cycle conditions were optimized as 10 s at 95°C, followed by forty cycles of amplification (5 s at 95 °C, and 30 s at 60 °C). Melt curve analysis following each real-time PCR was performed to assess product specificity. Each cDNA sample was subjected to quantitative RT-PCR analysis in triplicate. The Ct values of *Actin* gene are around 22 during kernel development. The standard curve of primers proved that the amplification efficiencies are nearly 100%. The results were analyzed by 2^−ΔΔCt^ method, and reported as mean ± SE (*n* = 3).

### 3.3. Vitamin E Extraction in Sweet Corn

Vitamin E from sweet corn was extracted according to the method reported, with some modifications [32]. In brief, 2 g of freshly ground sweet corn was saponified, firstly with 2 mL of ethanol (95%). 1 mL of sodium chloride (17.52 g/L), 4 mL of pyrogallol in ethanol (63.055 g/L), and 1 mL of ascorbic acid (176 mg/mL) were added as antioxidants. 2 mL of potassium hydroxide (600 g/L) was added, and mixtures were extracted with *n*-hexane/ethyl acetate (9:1 *v*/*v*). The organic layer was collected, and evaporated to dryness under nitrogen. After evaporation, residues were dissolved in *n*-hexane solution with isopropyl alcohol (1%) for HPLC analysis. Residues were also dissolved in acetone solution for the antioxidant activity quantification. The extracts were stored at −20 °C until analysis.

### 3.4. Quantification of the Vitamin E Isomers by NP-HPLC

The analysis of the sample extracts was completed by the normal phase HPLC reported previously [30]. The NP-HPLC method used a Breeze system for analysis, with a Waters 2475 Multi λ fluorescence detector and Waters 515 HPLC pump. An Agilent ZORBAX RX-SIL column (250 mm × 4.6 mm, and 5 μm particle size) was used, and the mobile phase was 0.85% (*v*/*v*) 2-propanol in *n*-hexane, with a flow rate of 1 mL/min. A 20 μL aliquot of sample was injected, and detected using an excitation wavelength of 290 nm and an emission wavelength of 330 nm. The quantitation of each vitamin E isomer in sample was achieved by comparing with eight external standards according to the retention time. α-, β-, γ-, δ-Tocopherols were purchased from Wako Pure Chemical Industries (Tokyo, Japan), and α-, β-, γ-, δ-tocotrienols were from Chromadex, Ltd. (Irvine, CA, USA). Each of the vitamin E isomer content was expressed as μg/100 g FW, and total vitamin E isomer content was the sum of four tocopherols and three tocotrienols from each sample. Data was reported as mean ± SD (*n* = 3).

### 3.5. Quantification of Lipophilic Antioxidant Activity

Lipophilic antioxidant activity was measured using lipophilic peroxyl radical scavenging capacity (Lipo-PSC) assay as reported previously [28]. The extracts in sweet corn were separated inthe same way as with vitamin E extraction, but the dry matter was dissolved inacetone for analysis. α-Tocopherol was used as standard. α-Tocopherol and the sample extracts were diluted into appropriate concentrations with 12% randomly methylated-β-cyclodextrin (RMCD) dissolved in 1:1 acetone/water solution. α-Tocopherol was prepared at a high concentration at first, and diluted into 2.0, 4.0, 8.0, 12.0, 16.0 μg/mL. 2,2′-Azobis-amidinopropane (ABAP) was used as radical donor, and 2,7-dichlorofluorescin diacetate (DCFH-DA) was used as fluorescence signal. Fluorescence generation was monitored (excitation at 485 nm and emission at 538 nm) by a Fluoroskan Ascent fluorescence spectrophotometer Molecular Devices F5 (Molecular Devices, Sunnyvale, CA, USA). Data were analyzed with the SoftMax^@^ Pro 6 software (Molecular Devices, LLC.). Results obtained for sample extracts antioxidant activities were expressed as μmol of α-tocopherol equiv./100 g FW. Data were reported as mean ± SD (*n* = 3).

### 3.6. Statistical Analysis

Statistical analyses were performed by Origin Pro 8 (Origin Lab Corporation, Northampton, MA, USA) and SigmaPlot software 12.3 (Systat Software, Inc., Chicago, IL, USA). Dose–effect analysis was performed using Calcusyn software version 2.0 (Biosoft, Cambridge, UK). Results were statistically analyzed among groups using one-way analysis of variance (ANOVA) and Ducan’s multiple comparison post-test. *p*-values less than 0.05 were regarded as statistically significant. Statistical analyses were performed using SPSS for Windows version 21.0 (SPSS Inc., Chicago, IL, USA). Correlation tests were performed using the Pearson’s correlation through SPSS 21.0.

## 4. Conclusions

In this study, we explored the expression levels of genes involved in vitamin E synthesis, the accumulation of vitamin E, and the antioxidant activity of sweet corn extracts. The expression levels of genes participated in vitamin E synthesis may affect the vitamin E contents during sweet corn kernel development.Both of expression levels of genes and the vitamin E contents were increased during the maturation stage. The lipophilic antioxidant activity of sweet corn extracts rose at 20–30 DAP in conjunction with the presence of vitamin E. In summary, vitamin E is a plant-derived antioxidant and beneficial compound to human body and animals. This study provided some information about the variation of vitamin E contents in sweet corn during the kernel development. The results may be beneficial for breeders to carry out to tochromanol bio-fortification in maize.

## Figures and Tables

**Table 1:** Variation of composition and contents ofvitamin E in sweet corn (μg/100 g in fresh weight).

	10 DAP	15 DAP	20 DAP	25 DAP	30 DAP
α-T	45.15 ± 1.46 ^e^	122.6 ± 2.9 ^d^	250.7 ± 2.4 ^c^	375.0 ± 18.9 ^b^	979.0 ± 55.6 ^a^
β-T	13.82 ± 0.04 ^e^	17.47 ± 0.12 ^d^	20.61 ± 0.05 ^c^	28.98 ± 0.81 ^b^	46.75 ± 2.08 ^a^
γ-T	29.29 ± 0.84 ^d^	91.79 ± 2.08 ^c^	115.6 ± 1.4 ^c^	272.3 ±12.4 ^b^	832.8 ±47.4 ^a^
δ-T	18.46 ± 0.09 ^d^	24.40 ± 0.32 ^c^	22.91 ± 0.10 ^c^	40.26 ± 1.10 ^b^	78.82 ± 3.62 ^a^
α-T3	94.07 ± 3.22 ^e^	168.4 ± 4.0 ^d^	205.7 ± 1.3 ^c^	253.2 ± 12.1 ^b^	383.6 ± 21.8 ^a^
γ-T3	115.2 ± 5.2 ^d^	728.7 ± 18.5 ^c^	758.8 ± 8.2 ^c^	992.1 ± 48.7 ^b^	1711 ± 100 ^a^
δ-T3	21.14 ± 0.18 ^d^	70.74 ± 1.27 ^b^	58.80 ± 0.60 ^c^	69.17 ± 2.39 ^b^	81.17 ± 3.94 ^a^
T/T3	0.46	0.26	0.40	0.55	0.89
α/γ-T	1.54	1.33	2.17	1.38	1.17
α/γ-T3	0.81	0.23	0.27	0.26	0.22

T = tocopherol; T3 = tocotrienol; α/γ = the ratio of total α-tocols/γ-tocols. Different superscript letters in each line indicate statistically significant differences between the means (*p* < 0.05) for each composition.

**Table 2:** Lipophilic antioxidant activity of sweet corn during days after pollination.

DAP	Concentration Range (mg/mL)	EC50 (mg/mL)	Dose Curve R^2^	Lipo-PSC Value (μmol α-Tocopherol Equiv./100 g FW)
10	80–400	NC *	NC	NC
15	50–400	NC	NC	NC
20	26.7–300	99.03 ± 20.48	0.964	39.08 ± 7.72 ^c^
25	20–200	63.08 ± 10.28	0.987	60.67 ± 9.10 ^b^
30	10–80	38.16 ± 8.01	0.981	101.8 ± 22.3 ^a^

* NC = no calculation results. Different superscript letters in Lipo-PSC Value column indicate statistically significant differences between the means (*p* < 0.05) for antioxidant activities.

**Table 3:** Real-time PCR primers of relative genes.

Gene Name	Gene ID	Prime Direction	Primer Sequence (5′-3′)
*_Zm_HPT*	732789	Forward	TCCATTGGCATCTGGGGAAT
		Reward	TGCAGTCCCAAGAACAAAGC
*_Zm_TC*	541877	Forward	TGGGATGGAGAACGGTTTGA
		Reward	CAGAAGCTCCTGGGAAGACA
*_Zm_TMT*	732837	Forward	CCATCATCACCTGTCGCAAG
		Reward	AGATGAGTAGACGGCGATGG
*_Zm_Actin*	100282267	Forward	TGTGGCTTTGGGATCGTAGTC
		Reward	GAGCCACCGATCCAGACACT
